# A multi-institutional phase II trial of bevacizumab for recurrent and refractory meningioma

**DOI:** 10.1093/noajnl/vdac123

**Published:** 2022-08-19

**Authors:** Priya Kumthekar, Sean Aaron Grimm, Roxanne T Aleman, Marc C Chamberlain, David Schiff, Patrick Y Wen, Fabio Massaiti Iwamoto, Demirkan Besim Gursel, David A Reardon, Benjamin Purow, Masha Kocherginski, Irene Helenowski, Jeffrey J Raizer

**Affiliations:** The Lou and Jean Malnati Brain Tumor Institute, Robert H. Lurie Comprehensive Cancer Center of Northwestern University, Chicago, IL, USA; Department of Neurology, Northwestern University, Feinberg School of Medicine, Chicago, IL, USA; Northwestern University, Chicago, IL, USA; Department of Neurology, Rush University Medical Center, Chicago, IL, USA; Department of Internal Medicine, Advocate Christ Medical Center, Oak Lawn, IL, USA; University of Washington, Fred Hutchinson Cancer Research Center, Seattle Cancer Care Alliance, Seattle, WA, USA; University of Virginia School of Medicine, Charlottesville, VA, USA; Dana-Farber Cancer Institute, Boston, MA, USA; Harvard School of Medicine, Boston, MA, USA; Columbia Medical Center, New York, NY, USA; Northwestern University, Chicago, IL, USA; Department of Pathology, Northwestern University, Feinberg School of Medicine, Chicago, IL, USA; Dana-Farber Cancer Institute, Boston, MA, USA; Harvard School of Medicine, Boston, MA, USA; University of Virginia School of Medicine, Charlottesville, VA, USA; Department of Preventative Medicine, Feinberg College of Medicine at Northwestern University, Chicago, IL, USA; Department of Preventative Medicine, Feinberg College of Medicine at Northwestern University, Chicago, IL, USA; The Lou and Jean Malnati Brain Tumor Institute, Robert H. Lurie Comprehensive Cancer Center of Northwestern University, Chicago, IL, USA; Department of Neurology, Northwestern University, Feinberg School of Medicine, Chicago, IL, USA; Northwestern University, Chicago, IL, USA

**Keywords:** anti-angiogenic, bevacizumab, dural tumors, hemangiopericytoma, high-grade meningioma, meningioma, solitary fibrous tumor

## Abstract

**Background:**

Systemic therapies for refractory meningiomas are limited with no FDA-approved therapeutics. Vascular endothelial growth factor (VEGF) is a signaling protein associated with neovascularization, peritumoral edema, and meningioma tumorigenesis.

**Methods:**

This phase II study investigates the efficacy of bevacizumab (BEV), a VEGF binding monoclonal antibody, in patients with progressive Grade I (G1M), Grade II (G2M), Grade III (G3M) meningioma, and other non-parenchymal tumors including vestibular schwannoma (*n* = 4) and hemangiopericytoma (*n* = 4) with the primary endpoint of progression-free survival rate at 6-months (PFS-6). Non-meningiomas were included with the respective meningioma grade in the analysis. Secondary endpoints include median overall survival (mOS) and response rate.

**Results:**

Fifty Patients (26 women; median age 54 years; range 23–81), 42 with progressive meningioma were treated: 10 G1M, 20 G2M, and 12 G3M. Prior treatments include surgical resection (41 patients), radiosurgery (24 patients), external beam radiotherapy (28 patients), and chemotherapy (14 patients). Median infusions administered were 16 (range, 2–68). Response was graded using the Macdonald’s criteria. PFS-6, median PFS, and mOS were 87%, 22 months, 35 months for G1M; 77%, 23 months, 41 months for G2M; and 46%, 8 months, 12 months for G3M. Best radiographic responses include stable disease (G1M: 100%; G2M: 85%; G3M: 82%); partial response (G1M: 0%; G2M: 5%; G3M: 0%) and progressive disease (G1M: 0%; G2M: 10%; G3M:18%). The most common toxicities were hypertension (*n* = 19, 42.2%), proteinuria (*n* = 16, 35.6%), and fatigue (*n* = 14, 31.1%).

**Conclusion:**

This study showed BEV is well tolerated and appears to be a promising systemic treatment option for patients with recurrent and refractory meningiomas.

**Key Points**

1. Bevacizumab is safe to use in patients with meningiomas, hemangiopericytomas, and vestibular schwannomas.
2. Bevacizumab may provide patients with a longer progression-free survival to disease treatment.

**Importance of the Study**

Meningiomas are the most common intracranial tumor. Standard-of-care includes surgical resection when possible and radiation therapy when indicated. Beyond surgery and radiation, recurrent and treatment-refractory meningiomas have no indication-specific FDA-approved systemic therapies and therefore these patients have very limited treatment options. This study is the largest prospective clinical trial study utilizing bevacizumab (BEV), a monoclonal antibody currently approved in the recurrent glioblastoma setting and known central nervous system safety profile. Patients on this study showed prolonged progression-free intervals in the setting of BEV use. This study could support larger clinical trials with bevacizumab in meningioma and support the use of BEV in patients with recurrent and refractory meningioma where there are very limited treatment options.

Meningiomas arise from neoplastic meningothelial arachnoid cap cells and represent approximately 35% of primary intracranial tumors in adults.^1,2^ Based on the degree of anaplasia, number of mitoses, presence of necrosis, and evidence of brain invasion, the World Health Organization (WHO) classifies meningiomas as benign (Grade I), atypical (Grade II), or malignant (Grade III).^3,4^

While asymptomatic meningiomas are typically managed through routine surveillance, the standard-of-care for patients exhibiting symptoms or tumor growth is gross total resection.^5^ Postoperative radiation therapy (RT), including external beam radiation therapy and stereotactic radiosurgery (RS), has largely been utilized as a safe and adjunct treatment for high-grade and recurrent low-grade meningiomas.^5–7^ However, a subset of patients receive systemic therapy due to disease progression following prior surgery or radiotherapy.^8^

The blood-brain barrier, which typically protects the brain and spinal cord from harmful substances, also prevents many forms of chemotherapy from entering the central nervous system (CNS). Although meningiomas develop outside of the blood-brain barrier and drug delivery is less of an issue, the currently available therapeutics have been largely inactive. Traditional cytotoxic chemotherapies act nonspecifically by damaging proliferating cells and therefore preferentially rapid cell cycling tumors. The majority of meningiomas, however, are slow growing and consequently, conventional chemotherapy exhibits limited efficacy^9,10^ As a result, treating aggressive, inoperable, or resistant meningiomas remains an unmet medical need.

Recent therapies have focused on targeting signaling pathways and growth factors thought to be important for meningioma growth and tumor angiogenesis. However, clinical trials on targeted molecular therapies suggest a lack of significant treatment response.^11–13^ To date, there are no FDA-approved systemic therapies for meningiomas.^14^ A recent meta-analysis of English language publications on systemic medical therapy for recurrent meningioma reported a progression-free survival rate at 6-months (PFS-6) of 29% for WHO Grade I meningiomas and 26% for combined WHO Grade II/III meningiomas.^12^ The authors propose that such results can be used to define a standardized endpoint and response criteria for treatment of recurrent meningiomas.

Vascular endothelial growth factor (VEGF) has been shown to play a significant role in neovascularization, tumor growth, and genesis of edema in meningiomas.^15^ Several studies have shown up-regulation of VEGF gene expression in CNS tumors as well as higher levels of VEGF mRNA, particularly in high-grade meningiomas.^16–20^ Prospective studies of vatalanib and sunitinib (oral inhibitors of VEGFR and other tyrosine kinases) demonstrate activity against recurrent Grade I and Grade II/III meningiomas as determined by meeting the PFS-6 benchmarks recommended by the Response Assessment in Neuro-Oncology (RANO) subcommittees.^21,22^ Bevacizumab (BEV) is a humanized VEGF ligand binding monoclonal antibody that is FDA-approved for the treatment of recurrent glioblastoma and several systemic malignancies. Retrospective studies of BEV for surgical and radiation-refractory meningiomas reported PFS-6 of 43.8% and 86%, suggesting therapeutic activity.^23,24^

The above-mentioned BEV data and relatively unfavorable toxicity profile of small molecule anti-angiogenic agents already studied, suggests that further studies are necessary to investigate the safety and efficacy of BEV in surgery and radiation-refractory meningioma. A prospective multicenter phase II trial was conducted to further assess the activity of BEV in patients with recurrent meningiomas where definitive surgery and RT were deemed not possible or already attempted.

## Methods

This single-arm phase II trial was conducted at Northwestern University, Washington University, Dana-Farber Cancer Center, Columbia University, and the University of Virginia from June 2010 to September 2013. Patients enrolled in the study signed institutional review board (IRB) approved informed consent form prior to registration. Patient characteristics, prior treatments, and treatment responses were recorded (Table 1). The primary tumor of interest was meningioma, but enrollment of hemangiopericytoma (HPC; also known as solitary fibrous tumor of the meninges), hemangioblastoma (HB), and acoustic/vestibular schwannoma (VS) patients were also accepted. The study was an investigator-initiated trial supported by funding from Genentech. The protocol was approved by all investigating site IRBs and informed consent was obtained from each participating patient.

**Table 1.** Patient Characteristics, Prior Treatment, and Treatment Response

Age	Sex	KPS (%)	WHO Grade	Diagnosis	Prior EBRT	Prior RS	Prior Surgeries	Prior Chemotherapy	No. Cycles	Best Response	PFS (m)	OS (m)
36	F	100	1	VS	0	0	1	N	8	PR	47.8	47.8
81	F	90	1	Men	2	0	0	N	5	SD	8.9	18.3
48	M	100	1	Men	0	1	1	N	2	SD	28.6	28.6
26	F	80	1	Men	1	3	6	N	4	SD	11.4	35
30	F	NA	1	Men	0	1	3	N	2	SD	22.5	26
32	F	80	1	VS	0	0	3	N	3	SD	24.7	24.7
49	F	90	1	VS	0	0	1	N	5	SD	32.8	32.8
23	M	90	1	VS	0	0	1	N	5	SD	32.5	32.5
80	M	80	1	Men	0	1	1	N	4	SD	16.8	36.1
63	F	80	1	Men	0	1	0	Sandostatin LAR	18	SD	47.4	47.4
65	M	80	1	Men	0	1	0	PTK787	4	SD	7.5	28.2
78	F	70	1	Men	1	0	0	N	5	SD	28.6	28.6
78	F	70	1	Men	1	0	0	N	3	SD	13.9	19.7
68	M	60	1	Men	1	1	2	N	2	PR	6	32.3
37	F	90	2	Men	0	3	2	N	7	SD	18.5	44.3
57	F	100	3	Men	0	1	4	Tamoxifen, HU	2	SD	2.7	5.9
52	F	70	2	Men	0	0	0	Sandostatin, HU	3	SD	5.9	25.8
63	M	NA	2	Men	1	1	1	N	NA	NA	1.3	1.3
49	F	70	2	Men	0	0	3	HU	1	PR	6.6	23.2
60	M	70	3	Men	2	2	5	HU	1	SD	9	9
53	M	70	3	Men	0	1	2	N	1	SD	19	19
79	M	70	2	Men	2	0	1	N	3	SD	9.5	26.9
41	M	90	3	Men	2	0	6	Y, unknown	1	SD	23.6	23.6
27	M	90	3	Men	2	0	3	Octreotide	20	SD	50.1	66
54	F	90	2	Men	1	0	2	N	12	PR	39.7	55.8
46	F	100	3	Men	1	0	3	N	23	SD	82.8	82.8
68	F	90	2	Men	0	0	0	HU, Sandostatin, Sunitinib, PTK 787, IFN-α	9	SD	23.4	58
57	M	60	3	Men	1	3	6	N	4	SD	8.2	51.3
45	M	80	3	Men	1	3	4	Hydroxyurea	1	SD	3.8	12.5
50	F	80	3	HPC	2	0	4	CAV	NA	PD	1.4	8.9
60	M	90	2	Men	1	0	4	HU, Sandostatin	16	SD	49.7	81.4
58	F	90	3	Men	1	1	0	N	1	SD	3.8	3.8
37	M	80	2	Men	0	3	2	N	7	SD	23.8	43.1
57	F	80	2	Men	2	3	4	HU	16	SD	40.8	40.8
57	M	100	2	Men	1	1	2	N	23	SD	65.8	72.4
45	F	80	2	HPC	1	0	3	N	7	SD	16.6	41.2
41	M	80	3	Men	1	1	1	N	8	SD	25.6	25.6
66	M	90	3	HPC	1	1	2	N	5	SD	11.7	11.7
61	M	NA	2	Men	1	1	3	N	1	SD	2.5	4
36	F	80	2	Men	1	0	3	N	22	SD	72.5	72.5
71	F	60	2	Men	0	0	9	N	1	SD	14.8	24.5
54	M	80	2	Men	0	0	2	N	3	SD	12.9	12.9
49	M	90	2	Men	0	0	0	N	11	SD	45	45
69	F	60	2	Men	1	0	1	N	2	SD	12.3	12.3
66	M	60	2	Men	1	0	3	N	8	SD	13	20.5
34	F	80	2	Men	0	1	3	N	5	SD	35.2	35.2
28	F	100	1	HPC	0	3	2	N	2	SD	6.2	29.9
46	M	70	3	Men	1	0	3	TMZ	1	PD	1.8	6.2
44	M	100	2	Men	0	2	2	N	6	SD	13.8	41.5
66	F	90	2	Men	1	0	2	N	4	SD	8.3	17.2

Abbreviations: EBRT, external beam radiotherapy; RS, radiosurgery; PFS, progression-free survival; OS, overall survival; GR, WHO Grade; HPC, hemangiopericytoma; N, none; SD, stable disease; Men, meningioma; VS, vestibular schwannoma; HU, hydroxyurea; TMZ, temozolomide; CAV, cyclophosphamide, Adriamycin, vincristine.

### Patient Eligibility

Patients were required to have a prior histologically proven meningioma, HPC, HB, or VS and have unequivocal radiographic evidence of tumor recurrence or progression. Patients with a history of neurofibromatosis type 2 were eligible if tumors that were not meningiomas or VS (ie, target lesions) were stable in size for the preceding six months. Recent resection was allowed if patients were greater than 4 weeks from surgery, had recovered from the effects of surgery, and had the residual evaluable disease. Prior treatment with other VEGF pathway inhibitors, except for BEV, was allowed. Patients were required to be ≥ 18 years, have a Karnofsky Performance Scale (KPS) ≥ 60%, and greater than a 12-week life expectancy. Patients were required to be more than 4 weeks from surgery, 8 weeks from RT, 4 weeks from cytotoxic chemotherapy, and 2 weeks from biologic therapy. The required initial laboratory values were an absolute neutrophil count ≥ 1000/mm^3^, platelets ≥ 100 000/mm^3^, hemoglobin ≥ 8 gm/dl, serum aspartate transaminase and serum alanine transaminase ≤ 3.5 × local laboratory upper limit of normal (ULN), creatinine ≤ 2.0 mg/dl, prothrombin time (PT)/partial thromboplastin time (PTT) ≤ 1.5 × ULN, total serum bilirubin ≤ 1.5 × ULN, and a urine protein: creatinine ratio ≤ 1.0 or the urine dipstick for proteinuria < 2. Anticoagulation with therapeutic warfarin (international normalized ratio (INR) < 3) or low molecular weight heparin was allowed.

Patients were not eligible for participation if there was a known hypersensitivity to BEV or a prior history of another malignancy (except nonmelanoma skin cancer or carcinoma in situ of the cervix) unless in complete remission and off all disease therapy for at least 5 years. Women of childbearing potential required a negative pregnancy test. Patients could not be pregnant and had to agree to contraception while on the study. Patients could not have any significant medical illnesses that were not adequately controlled or that would compromise the patient’s ability to tolerate BEV including any of the following: inadequately controlled blood pressure (defined as systolic blood pressure > 150 mmHg and/or diastolic blood pressure > 100 mmHg); history of hypertensive crisis or hypertensive encephalopathy; New York Heart Association Grade II or greater congestive heart failure; history of cardiac event within 12 months prior to starting treatment; history of cerebrovascular event within 6 months prior to registration; significant vascular disease within 6 months prior to starting treatment; evidence of bleeding diathesis within 28 days of starting treatment; history of major surgical procedure, open biopsy, or significant traumatic injury within 28 days of starting treatment; history of minor surgical procedure within 7 days of starting treatment; or history of abdominal fistula or gastrointestinal/bowel perforation within 6 months of starting treatment.

### Dosing and Scheduling of BEV

BEV was administered intravenously at a dose of 10 mg/kg over 90 min for the first dose then 30–60 min for remaining doses if no infusion reaction occurred on cycle 1 day 1. The drug was administered every 2 weeks for the first 6 months, after which patients were allowed to switch to every 3-week schedule at a dose of 15 mg/kg. Treatment continued until disease progression or intolerable side effects occurred. Premedication was allowed and at the discretion of the local institution’s standards for BEV infusions. One cycle was defined as 28 days on every 2-week schedule and 42 days on every 3-week schedule.

Contrast brain magnetic resonance imaging (MRI) was performed initially before starting treatment, and then again, every 8 weeks on an every 2-week schedule and every 12 weeks on an every 3-week schedule. Patients had a physical exam prior to starting treatment and then every 4 or 6 weeks depending on the infusion schedule. Proteinuria was monitored by urine protein, creatinine ratio, or dipstick every 6 weeks. If patients required elective major surgery, BEV was held 4–8 weeks prior to the procedure and were not allowed to restart BEV until 4 weeks after the procedure. A complete blood count and comprehensive metabolic panel were done on day 1 of every cycle and a 12-lead electrocardiogram (EKG) was performed at initial screening.

### Dose Modification

Modifications of the BEV dose were not allowed. If adverse events occurred requiring the withholding of treatment, the dose remained the same once treatment resumed. No more than 6 weeks were allowed between BEV doses. BEV was discontinued for patients with ≥ grade 2 pulmonary or CNS hemorrhage, ≥grade 3 non-pulmonary or non-CNS hemorrhage, congestive heart failure, grade 4 hypertension, proteinuria, and any grade arterial thrombotic event, gastrointestinal perforation, fistula, and reversible posterior leukoencephalopathy.

### Response Assessment

Objective responses were measured per the Macdonald criteria as the trial commenced before introduction of the RANO criteria.^12^ All measurements were performed on MRI T1W post-contrast images, with the largest cross-sectional area defining tumor size.

### Immunohistochemistry

*Immunohistochemistry* studies were performed on formalin-fixed, paraffin-embedded tissue sections to detect and evaluate the expression of VEGF, VEGFR2, and HER2. Sections were placed in 58–60°C oven for 60 min to increase the adherence of tissue to glass surface. All de-wax and antigen retrieval methods were executed by the Leica Bond-Max Autostainer. De-waxing was completed by using Leica Bond Dewax Solution (AR9222), followed by antigen retrieval with ER1 (Epitope Retrieval 1(AR9961) = PH 6 for 20 min). Based on the protocol for Leica Bond Polymer Refine Detection Kit (DS9800), the following incubation times applied for different steps: peroxide block for 5 min; primary antibody for 15 min; post-primary antibody for 8 min; Polymer HRP (secondary antibody) for 8 min; and substrate chromogen (DAB) for 10 min followed by hematoxylin. All slides were rehydrated through alcohol and xylene, mounted and cover slipped. Appropriate known control tissue was used for positive control and primary antibodies were omitted in negative controls. The following dilution and Leica Protocol reagents were used: VEGF (cat#ab39250 Abcam) Dilution 1:600 ER1(20) = Ph6 Leica Bond-Max protocol F, VEGFR2 (cat#2479 Cell signaling) Dilution 1:200 ER2(20) = Ph9 Leica Bond-Max protocol F, Her2 Dilution 1:1000 ER1(20) = Ph6 Leica Bond-Max protocol F. If there was any degree of positive VEGF2 staining, the sample was deemed “positive” and were categorized as low (1+), moderate (2+), and high (3+) VEGF. If no staining was seen, samples were scored as “negative.”

### Statistical Method

The primary endpoint was PFS-6 from the date of patient registration. PFS-6 rates and 95% confidence intervals were estimated using the Kaplan-Meier method. Initially, 20 patients were planned for each of the combined atypical/malignant and benign grades. For the atypical/malignant stratum, the null hypothesis of a 10% PFS-6 rate was tested against an alternative of 30%. It was determined that a sample size of 20 patients would be necessary to test this with 76% power. A sample size of 31 patients was included in this stratum. For the WHO Grade I stratum, the null hypothesis of a 50% PFS-6 rate was tested against an alternative of 80% and it was determined that a sample size of 20 patients would be necessary to test this with 80% power, however, only 14 grade 1 patients were enrolled and therefore a sample size of 14 patients was included in this stratum.

## Results

Fifty patients were enrolled in the trial between July 17, 2012 and September 18, 2013. There were 24 men and 26 women with a median age of 54 years (range 23–81) and median KPS of 80 (range 60–100). Accrual included: 14 WHO G1, 19 WHO G2, and 12 WHO G3. Of these 50 patients, 42 were diagnosed with meningioma: 10 G1M, 18 G2M, 10 G3M. The remaining 8 patients included 4 patients with recurrent/progressive VS and 4 patients with HPC (*n* = 1 grade 1, *n* = 1 grade 2, *n* = 2 grade 3). Patients had undergone prior treatments including surgical resection (median 2, range 1–9), RS (median 0, range 0–3), radiotherapy (median 1, range 1–2), and chemotherapy (median 4, range 1–5).

**Figure 1.**
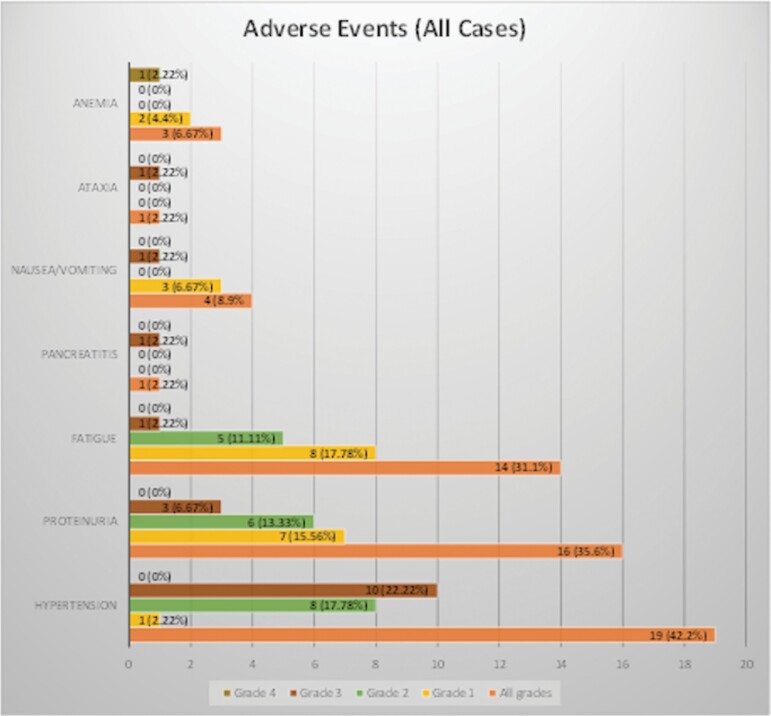
Bevacizumab treatment-related adverse events.

The median number of BEV infusions per patient was 16 (range 2–68). Nine patients changed from every 2-week to an every 3-week schedule following 6-months of stable disease. Although BEV was generally well tolerated, Grade 3 and 4 treatment-related toxicities did occur (Figure 1). The most common adverse events were hypertension (*n* = 19, 42.2%), proteinuria (*n* = 16, 35.6%) and fatigue (*n* = 14, 31.1%). At the time of data analysis, treatment was discontinued for progressive disease (*n* = 21, 42%), patient withdrawal (*n* = 10, 20%), toxicity (*n* = 7, 14%), surgical procedures (*n* = 2, 4%), patient death unrelated to treatment (*n* = 1, 2%), non-compliance (*n* = 1, 2%), and treating physician decision (*n* = 2, 4%). Six patients (12%) remain without progression at the time of the last data censorship in December 2020. Median follow-up for all patients was 31.9 months.

**Figure 2.**
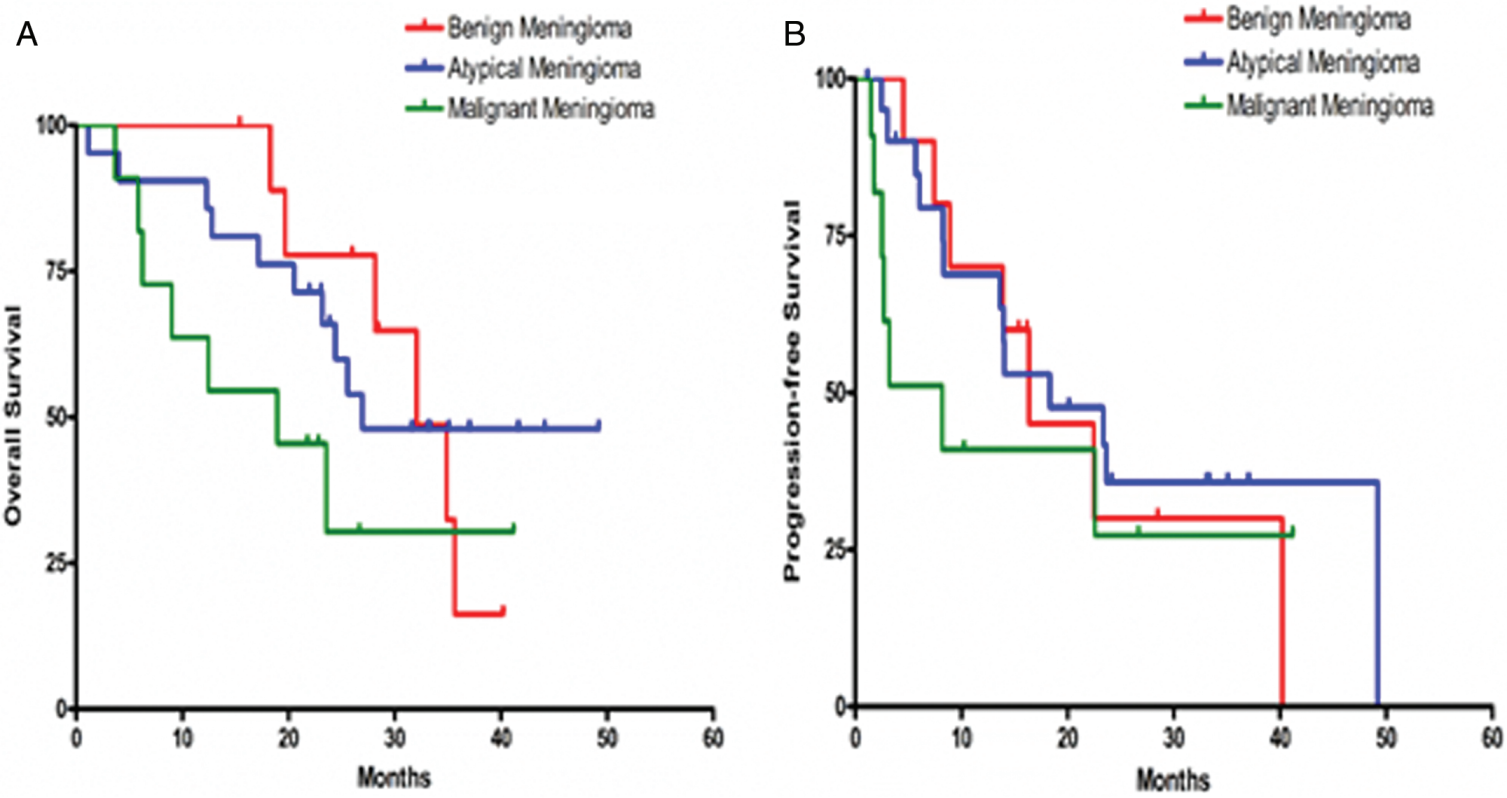
Kaplan-Meier curves for OS (A) and PFS (B) separated by Grade. PFS, progression-free survival; OS, overall survival.

**Figure 3.**
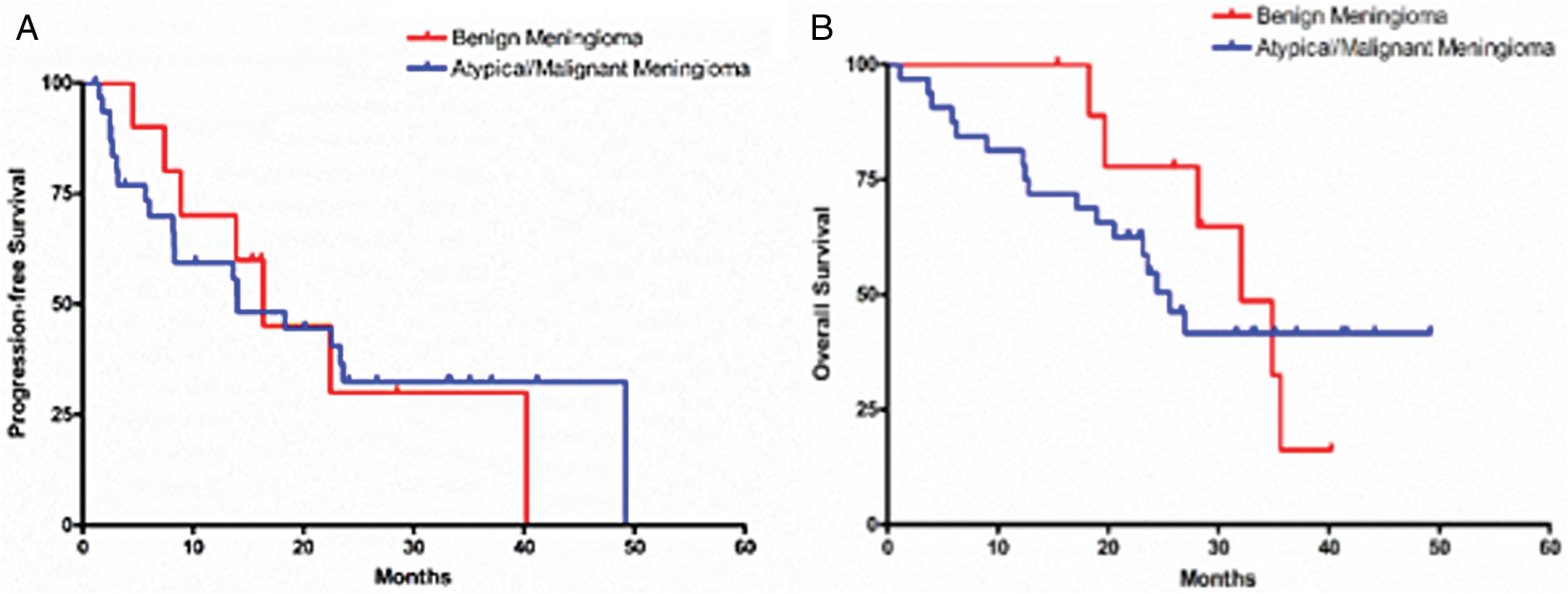
Kaplan–Meier curves for PFS (A) and OS (B) for WHO Grade I and WHO Grade II/III meningioma. WHO, World Health Organization; PFS, progression-free survival; OS, overall survival.

Progression-free survival at 6 months (PFS-6) was: 93% (61%, 99%) for WHO Grade I meningioma; 85% (61%, 95%) for WHO Grade II meningioma; 51% (22%, 75%) for WHO Grade III meningioma; and 73% (54%, 85%) for the combined group of WHO Grade II/III meningioma (Figure 2). PFS-6 for HPC and VS was 82% and 78%, respectively. Median PFS and OS were: 22 months and 35 months for WHO Grade I meningioma; 18 months and 27 months for Grade II meningioma; 8 months, and 12 months for WHO Grade III meningioma; and 14 months and 24 months for combined WHO Grade II/III meningioma (Table 2, Figures 2 and 3). Median PFS and OS were 18 and 35 months and 17 and 32 months for HPC and VS, respectively. The best radiographic response was stable disease in 86% of patients (Table 2). One patient (2%) with a Grade II meningioma had a partial response, as did one with VS. Four patients (8%) with WHO Grade II/III meningioma had progressive disease as the best radiographic response. In total there were 9 patients with negative VEGF staining, 29 patients were categorized as low VEGF2, 6 as moderate, and 1 as high VEGF2. There was no correlation between tumor grade and VEGF and VEGFR2 staining results. Further, in univariate and multivariate analysis, there was no correlation between VEGF/VEGFR2 expression and PFS-6.

**Table 2.** Median PFS, OS, and Best Response

Grade	PFS-6 (%)	mPFS (Months)	mOS (Months)	Best Response
Grade I meningioma (*n* = 10)	90	22	35	SD: 100% (*n* = 10)
Grade II/III meningiomas (*n* = 32)	66	14	24	
Grade II meningioma (*n* = 21)	76	18	27	SD: 85% (*n* = 19) PR: 5% (*n* = 1) PD: 10% (*n* = 2)
Grade III meningioma (*n* = 11)	45	8	12	SD: 82% (*n* = 9) PD: 18% (*n* = 2)

*Note*: Historical Benchmark PFS-6. Grade I—29% Grade II/II I—26%.

Abbreviations: PFS, progression-free survival; OS, overall survival; SD, stable disease.

## Discussion

Treatment options for surgical and radiation-refractory meningiomas remain limited. While higher-grade meningiomas are associated with poorer clinical outcomes, all grades of meningioma may result in significant morbidity as a consequence of tumor location and tumor-directed treatment.^25^ To date, there is no systemic medical therapy demonstrating prolonged PFS or overall survival in refractory meningiomas. Thus, there is no approved systemic therapy for this indication. The current study investigated the utility of BEV as a potentially promising therapy in this patient population.

Preclinical studies have demonstrated that VEGF is important for meningioma growth and proliferation.^26,27^ Retrospective and small prospective studies have suggested the potential benefit of VEGF pathway inhibitors for meningioma.^21–24,28^ Studies evaluating VEGF pathway inhibitors in recurrent WHO Grade II/III meningioma patients were reviewed (Table 3). The current study results suggest that BEV, a humanized VEGF ligand binding monoclonal antibody, improves PFS-6 in patients with refractory meningiomas as reflected by a 90% rate in Grade I and 66% rate in Grade II/III meningiomas. Both outcomes are superior to the historical benchmarks of 29% and 26% respectively, as determined by a recent meta-analysis.^12^ Notably, this meta-analysis was published approximately 5 years following the conception of this clinical trial and therefore the statistical benchmarks herein were not based on these values. Admittedly, it is difficult to compare prior chemotherapy and targeted therapy trials in recurrent meningioma as published reports are limited by small patient numbers, selection bias, inclusion of mixed histologic grades, and number of recurrences.^12^

**Table 3.** Studies Evaluating VEGF Pathway Inhibitors in Recurrent WHO Grade II/III Meningioma

References	Treatment	No. Patients	Grade	mPFS (m)	OS (m)	PFS-6 (%)
{Kaley} (P)	Sunitinib	28	II/III	4.6		36
{Lou}(R)	Bevacizumab ± TMZ or VP-16	4 8	I II/III	12.2 15.8		80 87.5
{Nayak} (R)	Bevacizumab	15	II/III	26	15	43.8
{Raizer} (P)	Valatanib	22	II/III	7.0	26	54.4
{Shih) (P)	Bevacizumab + everolimus	4 12	I II/III	17.5 22	23.8 (All)	69 (All)
Current study	Bevacizumab	10	I	22	35	90
		21	II	18	27	76
		11	III	8	12	45
		32	II/III	14	24	66

Abbreviations: PFS, progression-free survival; m, months; OS, overall survival; P, prospective; R, retrospective; TMZ, temozolomide; VP-16, etoposide; WHO, World Health Organization; VEGF, vascular endothelial growth factor.

The current trial was a single-arm phase II study stratified by meningioma grade per the traditional WHO classification.^3^ Outcomes of Grade II and III patients were in addition analyzed separately for descriptive purposes. The study results suggest BEV is an active agent and should be considered for use in patients with refractory meningiomas. The current study prospectively confirms the benefit of BEV relative to other targeted and chemotherapeutic agents that have been utilized to date. The study also demonstrated that while PFS can be prolonged, this benefit occurs with radiographic stable disease given that objective radiographic response is rare.

Limitations of this study include a relatively small sample size and variability in both meningioma tumor grade and previous therapies completed. These prior treatments included partial or complete surgical resection (41 patients), RS (24 patients), external beam radiotherapy (28 patients), and chemotherapy (14 patients). Nonetheless, this trial includes a larger study population than most prior published trials. Furthermore, the trial utilized a single unblinded treatment arm without a control group. However, as there is no standard therapy, designing such a trial is challenging to execute. Meningiomas also have a variable growth rate, making it difficult to measure treatment effect with consistency. This is particularly relevant in that eligibility criteria required evidence of radiographic progression prior to entering the study, though no firm definition regarding pre-BEV treatment progression was included. More recently and to ensure homogeneity of patients upon study entry, criteria for pretreatment disease progression have been suggested.^29^ As this study was planned and opened before these criteria were published, a specific rate of growth pre-study registration was not captured. Furthermore, as radiographic assessment indicated primarily stable disease, a trial design assessing the duration of stable disease with BEV treatment could potentially better define the role of BEV in treating recurrent meningioma. Lastly, response assessment utilized the Macdonald criteria and not the revised RANO criteria, and therefore fluid-attenuated inversion recovery (FLAIR) changes in MRI tumor size were not considered as radiographic endpoints—though likely this is a minor issue with meningiomas.^30^ Further, as objective radiographic responses were rare, pseudoresponse—wherein a decrease in contrast enhancement occurs without an objective change in overall tumor volume, often seen with anti-angiogenic agents—did not confound the current study results.

In conclusion, despite the study limitations discussed, BEV appears to result in prolonged stability for patients with recurrent and treatment-refractory meningiomas. Once surgical and RT options have been trialed, BEV may be considered as a potential next-line therapy. A larger clinical trial should also be considered to further investigate the efficacy of BEV.

## References

[1] Huntoon K , Toland AMS, Dahiya S. Meningioma: a review of clinicopathological and molecular aspects. Front Oncol. 2020;10(October):1–14. 33194703 10.3389/fonc.2020.579599 PMC7645220

[2] Ostrom QT , Gittleman H, Fulop J, et al CBTRUS statistical report: primary brain and central nervous system tumors diagnosed in the United States in 2008–2012. Neuro Oncol. 2015;17(suppl 4 ):iv1–iv62. 26511214 10.1093/neuonc/nov189 PMC4623240

[3] Louis DN , Perry A, Reifenberger G, et al The 2016 world health organization classification of tumors of the central nervous system: a summary. Acta Neuropathol. 2016;131(6):803–820. 27157931 10.1007/s00401-016-1545-1

[4] Commins DL , Atkinson RD, Burnett ME. Review of meningioma histopathology. Neurosurg Focus. 2007;23(4):1–9. 17961040 10.3171/FOC-07/10/E3

[5] Aizer AA , Arvold ND, Catalano P, et al Adjuvant radiation therapy, local recurrence, and the need for salvage therapy in atypical meningioma. Neuro Oncol. 2014;16(11):1547–1553. 24891451 10.1093/neuonc/nou098 PMC4201070

[6] Brastianos PK , Galanis E, Butowski N, et al Advances in multidisciplinary therapy for meningiomas. Neuro Oncol 2019;21(suppl 1): I18–I31. 30649489 10.1093/neuonc/noy136 PMC6347080

[7] Debus J , Wuendrich M, Pirzkall A, et al High efficacy of fractionated stereotactic radiotherapy of large base-of-skull meningiomas: long-term results. J Clin Oncol. 2001;19(15):3547–3553. 11481362 10.1200/JCO.2001.19.15.3547

[8] Norden AD , Drappatz J, Wen PY. Advances in meningioma therapy. Curr Neurol Neurosci Rep. 2009;9(3):231–240. 19348712 10.1007/s11910-009-0034-5

[9] Al-Rashed M , Foshay K, Abedalthagafi M. Recent advances in meningioma immunogenetics. Front Oncol. 2020;9(January):1–11. 10.3389/fonc.2019.01472 PMC6960175 31970090

[10] Choy WC , Kim W, Nagasawa D, et al The molecular genetics and tumor pathogenesis of meningiomas and the future directions of meningioma treatments. Neurosurg Focus. 2011;30(5):E6. 10.3171/2011.2.FOCUS1116 21529177

[11] Wen PY , Quant E, Drappatz J, Beroukhim R, Norden AD. Medical therapies for meningiomas. J Neurooncol. 2010;99(3):365–378. 20820875 10.1007/s11060-010-0349-8

[12] Kaley T , Barani I, Chamberlain M, et al Historical benchmarks for medical therapy trials in surgery-and radiation-refractory meningioma: a RANO review. Neuro Oncol. 2014;16(6):829–840. 24500419 10.1093/neuonc/not330 PMC4022224

[13] Sherman WJ , Raizer JJ. Chemotherapy: what is its role in meningioma? Expert Rev Neurother. 2012;12(10):1189–95; quiz 1196. 23082735 10.1586/ern.12.108

[14] Wen PY , Drappatz J. Novel therapies for meningiomas. Expert Rev Neurother. 2006;6(10):1447–1464. 17078786 10.1586/14737175.6.10.1447

[15] Machein MR , Plate KH. VEGF in brain tumors. J Neurooncol. 2000;50(1–2):109–120. 11245271 10.1023/a:1006416003964

[16] Berkman RA , Merrill MJ, Reinhold WC, et al Expression of the vascular permeability factor/vascular endothelial growth factor gene in central nervous system neoplasms. J Clin Invest. 1993;91(1):153–159. 8380810 10.1172/JCI116165 PMC330009

[17] Christov C , Lechapt-Zalcman E, Adle-Biassette H, Nachev S, Gherardi RK. Vascular permeability factor/vascular endothelial growth factor (VPF/VEGF) and its receptor flt-1 in microcystic meningiomas. Acta Neuropathol. 1999;98(4):414–420. 10502048 10.1007/s004010051102

[18] Samoto K , Ikezaki K, Ono M, Shono T, Kuwano M. Expression of vascular endothelial growth factor and its possible relation with neovascularization in human brain tumors. Cancer Res. 1995;55(5):1189–1193. 7532545

[19] Lee SH , Lee YS, Hong YG, Kang CS. Significance of COX-2 and VEGF expression in histopathologic grading and invasiveness of meningiomas. J Pathol Microbiol Immunol. 2013;122(1):16–24. 10.1111/apm.12079 23756256

[20] Reszec J , Hermanowicz A, Rutkowski R, Turek G, Mariak Z, Chyczewski L. Expression of MMP-9 and VEGF in meningiomas and their correlation with peritumoral brain edema. Biomed Res Int. 2015; 2015:646853. 25821815 10.1155/2015/646853 PMC4363610

[21] Raizer JJ , Grimm SA, Rademaker A, et al A phase II trial of PTK787/ZK 222584 in recurrent or progressive radiation and surgery refractory meningiomas. J Neurooncol. 2014;117(1):93–101. 24449400 10.1007/s11060-014-1358-9

[22] Kaley TJ , Wen P, Schiff D, et al Phase II trial of sunitinib for recurrent and progressive atypical and anaplastic meningioma. Neuro Oncol. 2015;17(1):116–121. 25100872 10.1093/neuonc/nou148 PMC4483051

[23] Nayak L , Iwamoto FM, Rudnik JD, et al Atypical and anaplastic meningiomas with bevacizumab. J Neurooncol. 2012;109(1):187–193. 22544653 10.1007/s11060-012-0886-4

[24] Lou E , Sumrall AL, Turner S, et al Bevacizumab therapy for adults with recurrent/progressive meningioma: a retrospective series. J Neurooncol. 2012;109(1):63–70. 22535433 10.1007/s11060-012-0861-0 PMC3404217

[25] Nakasu S , Notsu A, Na K, Nakasu Y. Malignant transformation of WHO grade I meningiomas after surgery or radiosurgery: systematic review and meta-analysis of observational studies. Neuro Oncol Adv. 2020;2(1):1–12. 10.1093/noajnl/vdaa129 PMC7712809 33305267

[26] Pietsch T , Valter M, Wolf H, et al Expression and distribution of vascular endothelial growth factor protein in human brain tumors. Acta Neuropathol. 1997;93(2):109–117. 9039457 10.1007/s004010050591

[27] Nishikawa R , Cheng SY, Nagashima R. Expression of vascular endothelial growth factor in human brain tumors. Acta Neuropathol. 1998;96(5):453–462. 9829808 10.1007/s004010050919

[28] Shih KC , Chowdhary S, Rosenblatt P, Weir AB III, Shepard GC. A phase II trial of bevacizumab and everolimus as treatment for patients with refractory, progressive intracranial meningioma. J Neurooncol. 2016;129(2):281–288. 27311730 10.1007/s11060-016-2172-3

[29] Huang RY , Bi WL, Weller M, et al Proposed response assessment and endpoints for meningioma clinical trials: report from the response assessment in Neuro-Oncology Working Group. Neuro Oncol. 2019;21(1):26–36. 30137421 10.1093/neuonc/noy137 PMC6303427

[30] Enokizono M , Morikawa M, Matsuo T, Hayashi T. The rim pattern of meningioma on 3D FLAIR Imaging: correlation with tumor-brain adhesion and histological grading. Magn Reson Med Sci. 2014;13(4):251–260. 25167879 10.2463/mrms.2013-0132

